# Case analysis and finite element analysis of adult head skull fractures in people run over by motor vehicles

**DOI:** 10.1093/fsr/owaf007

**Published:** 2025-02-17

**Authors:** Xingan Yang, Xinzhe Chen, Fu Zhang, Tengfei Yang, Jiangwei Kong, Xinbiao Liao, Dongri Li

**Affiliations:** Department of Forensic Pathology, School of Forensic Medicine, Southern Medical University, Guangzhou, China; Guangdong Provincial Key Laboratory of Automotive Engineering, South China University of Technology, Guangzhou, China; Guangdong Public Security Department, Forensic Pathology Lab, Guangzhou, China; Department of Forensic Pathology, School of Forensic Medicine, Southern Medical University, Guangzhou, China; Department of Forensic Pathology, School of Forensic Medicine, Southern Medical University, Guangzhou, China; Guangdong Public Security Department, Forensic Pathology Lab, Guangzhou, China; Department of Forensic Pathology, School of Forensic Medicine, Southern Medical University, Guangzhou, China

**Keywords:** forensic pathology, run-over injury, skull fracture, finite element analysis

## Abstract

The cranial stress distribution and morphological characteristics of cranial fractures in adult heads run over by motor vehicle were investigated. Cases of adults’ heads run over by motor vehicles were collected. The skull fracture degrees are categorized into no skull fracture, multiple skull fractures, and skull fractures with brain tissue overflow. According to China’s road traffic management system, vehicles in traffic accidents are categorized as heavy vehicles (weight ≥ 12 t), medium vehicles (4.5 t ≤weight < 12 t), light vehicles (1.8 t <weight < 4.5 t), and light minicars (weight ≤ 1.8 t). Finite element simulations were used to analyze the relationship between cranial fracture morphology and vehicle weight as well as cranial stress response. A total of 41 cases of heads run over by motor vehicles were collected. In seven cases of multiple skull fractures caused by medium vehicles, there were skull fractures on both the ground side and the tyre side, and the degree of skull fracture was more serious on the ground side than on the tyre side. Finite element simulations showed that the cranial stress in was more concentrated on the ground side than on the tyre side. Light vehicles and light minicars running over a head usually do not fracture the skull. Head injuries caused by medium vehicles are mainly characterized by multiple skull fractures, and the degree of skull fracture is more serious on the ground side than on the tyre side.

## Introduction

Traffic accidents in which human bodies are run over by motor vehicles are relatively rare, and there have been few related studies, but these accidents can easily cause disputes due to the complexity of the case and the high mortality rate. Hitosugi et al. [1] investigated and studied road traffic accidents in Japan from 2012 to 2016, and proposed that 8.3% of traffic accidents involved pedestrians being run over, and 4% of the run over cases involved hit-and-run. However, there are only sporadic reports on the causes of traffic accidents about running over, without statistical induction. Szleszkowski et al. [2] reported on a man who was run over by a truck whilst crossing a crosswalk, and Schrag et al. [3] reported a traffic accident in which a woman walking on a highway was hit by a truck and then run over by a second car. Takeshi et al. [4] reported seven cases of children being run over in traffic accidents, six of which occurred in parking lots or garages. Doro et al. [5] reported two cases of adults being run over because they lay in the middle of the road, one of which was the suicidal behaviour of a mentally ill woman, and the other was run over because he slept drunk on the road. Buck et al. [6] reported three cases of being run over by motor vehicles, and they used 3D reconstruction technology to explore the causes of accidents. In addition to determining whether the run over occurred and the cause of death, forensic pathologists usually need to determine the cause of the run over based on the facts of the case, a site investigation and a vehicle investigation. In traffic accidents involving running over, head running over is special to some extent, especially in secondary accidents, forensic pathologists need to determine whether fatal craniocerebral injury has been caused at the time of the first running over [3, 6]. It is also important to note that a fatal head injury can occur during a seizure or during a drunken fall, so a forensic pathologist needs to carefully examine the head injury and it is important to identify whether it was caused by running over or by other means [5]. The tolerance of skull fractures caused by running over is very important in the identification of skull fractures due to running over or other forms of injury.

To explore the tolerance limit of skull fractures, Yoganandan and Pintar [7] conducted quasi-static compression tests with 13 unembalmed human heads collected as early as 1880 and reported that the tolerance limit of skull fractures was 400–600 kg for men and 300–800 kg for women. Yoganandan et al. [8] conducted skull fracture experiments on 12 unembalmed human cadaver heads by using electrohydraulic testing devices. The loading surface was a hemispherical sphere with a radius of 48 mm, and it was found that an average static load of 6.4 kN was required to cause a skull fracture. The load range for the linear fractures of temporal, parietal and zygomatic bones was 4.464 kN–5.915 kN. De Kegel et al. [9], using the finite element head model of the University of Strasbourg (SUFEHM), showed that the energy threshold for 50% skull fracture risk ranged from 453 mJ to 833 mJ. However, skull fracture not only depends on the force but also has a close correlation with the contact area, action site and action time of the injured object.

Therefore, this study collected traffic accident cases of adults whose head was run over by motor vehicles, and summarized the causes of the accidents. The accident vehicles and the tyres that run over the head were weighed, the degree of skull fracture was determined, and combined with the finite element simulation technology, the tolerance and morphological characteristics of skull fracture caused by running over were analyzed and discussed.

## Materials and methods

### Cases

In every case, the trunk of the individual was run over by a vehicle first, then the left and right temporal parts of the head were run over, and the vehicle speed was ˂40 km/h. In each situation, an on-site inspection, an accident vehicle inspection, and a systematic forensic pathological anatomy examination were performed. According to the systematic anatomy assessment, the thickness of the skull (frontal bone, temporal bone and occipital bone) was required to meet the reference value of the adult skull thickness in a previous study [10]. The thickness of frontal bone, parietal bone and occipital bone was 6.66 mm ± 1.50 mm, 5.43 mm ± 1.04 mm, 7.47 mm ± 2.31 mm for males, and 7.44 mm ± 1.65 mm, 5.58 ± 1.04 mm, 8.26 ± 2.65 mm for females [10]. Sex, age, vehicle type, speed, cause of falling, cause of death, morphology, and degree of skull fracture were summarized and sorted. According to the public safety industry standard GA 802–2019 road traffic management on vehicle types of the People’s Republic of China [11], the vehicles involved in the accident were classified as follows: heavy vehicles (weight ≥ 12 t); medium vehicles (4.5 t ≤ weight < 12 t); light vehicles (1.8 t < weight < 4.5 t); and light minicars (weight ≤ 1.8 t). According to the autopsy, the causes of death and injury were determined, and the degrees of skull fracture were categorized into three grades: I (no skull fracture); II (multiple skull fractures); and III (skull fractures with brain tissue overflow). For the case of multiple skull fractures, after identifying the tyre side and the ground side by surveillance videos and systematic inspection, the fracture degree of the tyre side and the ground side of each case was compared according to the number of fracture lines.

### Simulation conditions and methods

#### Finite element (FE) model

In this study we used the FE head model constructed by Ma et al. [12]. The geometry of the head model was established using computed tomography (General Electric multi-spiral CT, Model number: LightSpeed OX/I) and magnetic resonance tomography (GE Signa EXCITE HD 1.5 T), based on data from Chinese males at the 50th percentile (height: 1 678.0 ± 59.93 mm, weight: 59.0 ± 6.66 kg). FE head model could predict head responses, however, the performance and biofidelity of the model depend on correct anatomical structure and material description of these structures and mesh quality [9, 13–15]. In the study, the model mainly includes a scalp, a skull and a facial bone. The skull is subdivided into the brain, cerebellum, brainstem, falx cerebellum, tentorium cerebellum, callosum’s body, dura mater, pia mater, and cerebrospinal fluid. The model was mainly divided by hexahedral grid elements, with 96 733 solid elements, 69 599 shell elements and a 4.52 kg skull mass. Compared with other head models, this study used the FE head model constructed by Ma et al. [12], who improved the head anatomical structure, the mesh quality and the material of some head tissues, especially the material of the cerebrospinal fluid and the coupling relationship amongst the skull, brain and cerebrospinal fluid. The FE model of the head has demonstrated the ability to reflect impact damage and a high degree of imitation [12]. At present, the main material of most automobile tyres is rubber, with natural rubber, butadiene rubber, styrene butadiene rubber, and butyl rubber as the most widely used. In this study, in order to simulate the real tyre texture, we adopted ultra-elastic rubber material to establish the tyre model. The width of the tyre is 200 mm, the diameter is 15 in, that is, 3 810 mm, and the section height is 107 mm.

#### Simulation methods

**Figure 1:**
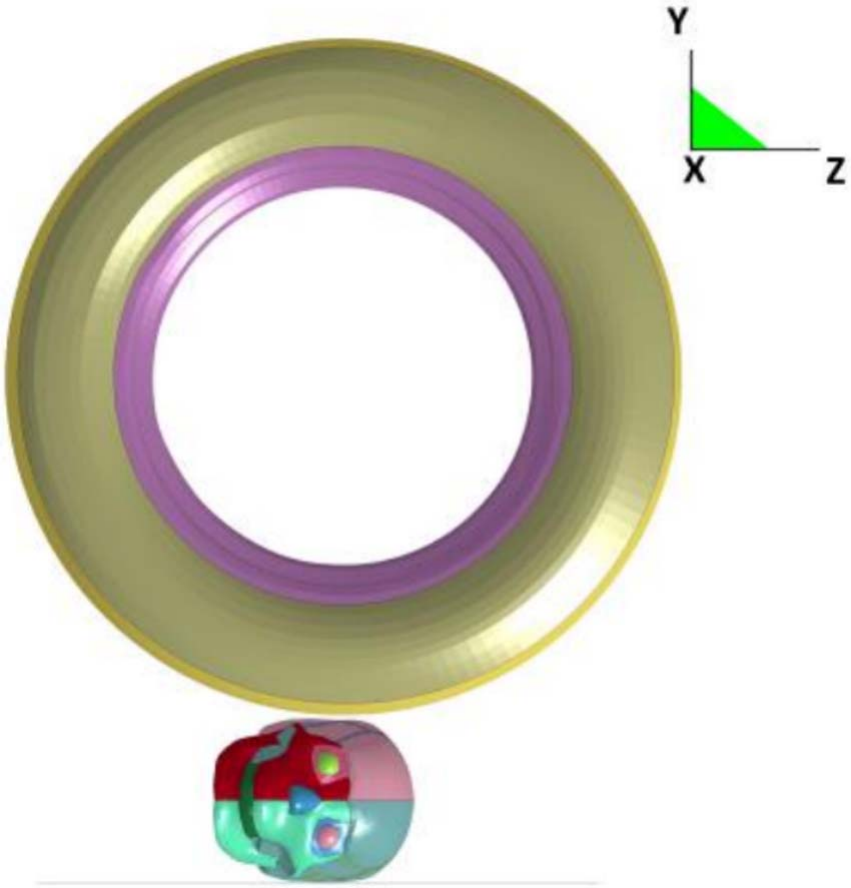
The wheels apply a vertical downward force, reducing the ground to a fixed square surface with sides of 300 mm.

**Figure 2:**
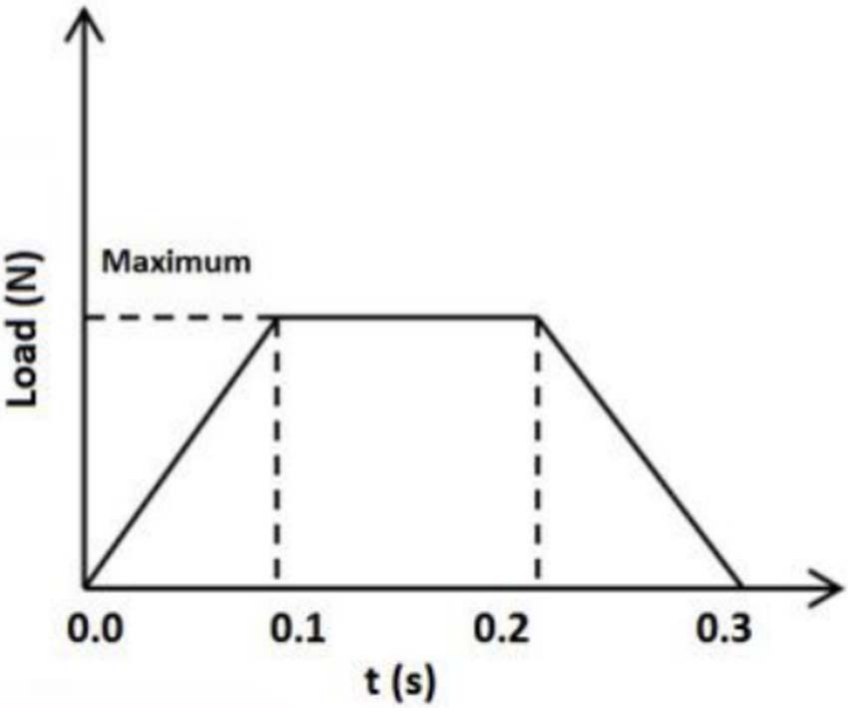
According to the speed of all the accidents, i.e. below 40 km/h, the simulation speed is set as 10 m/s (36 km/h), and the wheel running over the head time is 0.3 s. The load of the tyre vertically downward on the head increases from small to large and then decreases gradually after a period of stability to simulate the situation of the tyre passing through the head.

In an actual traffic accident, when a wheel runs over the head of an individual, the force loaded on the head increases and then decreases. Therefore, to simplify the simulation mode, the wheel is loaded with vertical downward force, and the ground below is simplified into a square fixed surface with a side length of 300 mm (Figure 1). According to the speed of all the accidents, i.e. below 40 km/h, the simulation speed is set as 10 m/s (36 km/h), and the wheel running over the head time is 0.3 s. According to the weight given to the ground by the tyres of different models that crush the skull, the load assigned to the simulation tyres is set as 1 000 kg, 1 500 kg, and 2 000 kg. The load of the tyre vertically downward on the head increases from small to large and then decreases gradually after a period of stability to simulate the situation of the tyre passing through the head (Figure 2).

## Results

### Vehicle type, sex, and age

A total of 41 cases were examined, including 26 males and 15 females aged 18–85 years (Table 1). Accident vehicles include 19 heavy vehicles, *n* = 19; nine medium vehicles, *n* = 9; six light vehicles, *n* = 6; and seven light minicars, *n* = 7. Thirty-nine of the 41 accidents occurred on urban roads (general speed limit <40 km/h), one accident occurred in a residential area (speed <10 km/h), and one occurred in a parking lot (speed <10 km/h).

**Table 1:** Statistics on sex and age.

Type of fractures	Men	Women	Average age (years)	Minimum age (years)	Maximum age (years)	Total
I	4	7	42.64	28	82	11
II	8	0	43.00	32	67	8
III	14	8	43.36	18	85	22
Total	26	15	43.10	18	85	41

I: no skull fracture; II: multiple skull fractures; III: skull fractures with brain tissue overflow.

### Causes for running over and other relevant information

The causes of running over can be classified into five types (Table 2). Type A: in a total of 15 cases, riders were run over by other vehicles after colliding with them whilst travelling on a road on either a motorcycle or bicycle. The vehicle speed was ˂40 km/h. Amongst these cases, there were five hit-and-run incidents, accounting for 33.3%. Type B: in a total of 11 cases, heavy or medium vehicles, knocked down pedestrians, bicycles or motorcycles when turning right, and then ran over them. The vehicle speed was ˂10 km/h. Amongst these cases, there were two hit-and-run incidents, accounting for 18.2%. Type C: in a total of eight cases, individuals fell to the ground as a result of drunk driving on bicycles or motorcycles and were subsequently run over by a vehicle (including bicycles, *n* = 5; motorcycles, *n* = 1; pedestrians, *n* = 2). The vehicles’ speed was ˂40 km/h. Amongst these cases, there were four hit-and-run incidents, accounting for 50%. Type D: in a total of five cases, pedestrians, bicycles, or motorcycles were hit by either a heavy or medium vehicle at an intersection, and then the pedestrians or riders were run over. The vehicles’ speed was ˂10 km/h in these cases, and there were no hit-and-run incidents. Type E: there were two cases of individuals being run over as a result of their own illnesses. In one case, the individual experienced a sudden cerebral haemorrhage, fell to the ground and was run over by a reversing light vehicle. The vehicle speed was ˂10 km/h. The driver did not flee. In another case, a patient with schizophrenia was run over by a light minicar whilst sleeping on the road at night. The vehicle speed was ˂40 km/h. The driver fled.

**Table 2:** Causes of running over and other relevant information.

Causes of running over	Amount (*n*, %)	Speed (km/h)	Hit-and-run incidents (*n*, %)
A	15(36.6)	<40	5(33.3)
B	11(26.8)	<10	2(18.2)
C	8(19.5)	<40	4(50.0)
D	5(12.2)	<10	0(0)
E	2(4.9)	<10	1(50.0)

A: riders were run over by other vehicles after colliding with them whilst travelling on a road on either a motorcycle or bicycle; B: heavy or medium vehicles, knocked down pedestrians, bicycles or motorcycles when turning right, and then ran over them; C: fell down drunk and was run over; D: pedestrians, bicycles, or motorcycles were hit by either a heavy or medium vehicle at an intersection, and then the pedestrians or riders were run over; E: illnesses.

### Vehicle type, head injury, and cause of death

There were 19 cases of heavy vehicles running over individuals. All skull fractures were type III, and all individuals died instantly due to severe damage to multiple organs. The weight of the tyre on the head of the individual during running over was ˃3 000 kg. There were nine cases of medium vehicles running over individuals. All individuals died instantly due to severe damage to multiple organs. Two cases of skull fractures were type III, and the tyres that run over the heads exerted a weight of 2 444 kg and 2 333 kg when on a weighbridge, respectively. Actual force may vary at the time of the accident. The other seven cases were type II, and the weight of the tyre ranged from 1 013 kg to 1 669 kg when the vehicle ran over the head. There were six cases of light vehicles running over individuals. Five cases of skull fractures were type I. The weight of the tyre on the head of the individual during running over was ranged from 611 kg to 941 kg. Two of the five patients died at the scene, and the causes of death were heart rupture and cardiopulmonary contusion. The other three patients died of traumatic shock caused by liver and spleen failure. In the last case, an individual who was drunk and out of control fell to the ground resulting in a fractured skull whilst riding a bicycle, and then was run over. The cause of death was severe damage to multiple organs, and the skull fracture was classified as type III. The weight of the vehicle was 3.34 t, and the weight of the tyre on the individual’s head whilst running over was 982 kg. There were seven cases of light minicar running over individuals. Six cases of skull fractures were type I. The weight of the tyre on the head of the individual during running over ranged from 324 kg to 444 kg. The cause of death was traumatic shock in three cases. In one case, the individual suffered a cardiopulmonary contusion. In one case of bilateral lung contusion, a patient with acute pulmonary thromboembolism was identified. Another individual suffered a skull fracture caused by falling off of a bicycle during drunk cycling and then was crushed by a vehicle. The skull was fractured multiply, but the brain tissue did not overflow, and the cause of death was severe damage to multiple organs. The weight of light minicar was 1.45 t, and the weight of the tyre on the head of the individual during running over was 426 kg (Tables 3 and 4).

**Table 3:** Statistical table on the types of cranial fractures, vehicle types, and tyre weights when a head is run over.

Vehicle type	Downwards force on tyre, measured on weighbridge	Type and number of fractures			Total
		I	II	III	
Heavy vehicle	>3 000 kg	0	0	19	19
Medium vehicle	1 013–2 444 kg	0	7	2	9
Light vehicle	611–982 kg	5	0	1^a^	6
Light minicar	324–444 kg	6	1^a^	0	7
Total	–	11	8	22	41

a The presence of skull fracture before running over, which is determined by systematic anatomy combined with on-site and accident vehicle inspection; heavy vehicles (weight ≥ 12 t), medium vehicles (4.5 t ≤weight < 12 t), light vehicles (1.8 t <weight < 4.5 t), and light minicars (weight ≤ 1.8 t).

I: no skull fracture; II: multiple skull fractures; III: skull fractures with brain tissue overflow.

**Table 4:** Causes of death and types of vehicles.

Vehicle types	Causes of death				Total
	Damage to multiple organs	Cardiopulmonary contusion	Traumatic shock	Pulmonary thromboembolism	
Heavy vehicle	19	0	0	0	19
Medium vehicle	9	0	0	0	9
Light vehicle	1^a^	2	3	0	6
Light minicar	1^a^	2	3	1	7
Total	30	4	6	1	41

Traumatic shock is mainly caused by the rupture of the liver and spleen.

a The presence of a skull fracture before running over, which is determined by systematic anatomy combined with accident scene and vehicle inspection.

In seven cases of multiple skull fractures caused by medium vehicles, fractures were found in both on the ground side and on the tyre side of the skull, and the number of fracture lines on the ground side of the skull was significantly greater than that on the tyre side of the skull in each case.

### FE analysis

**Figure 3:**
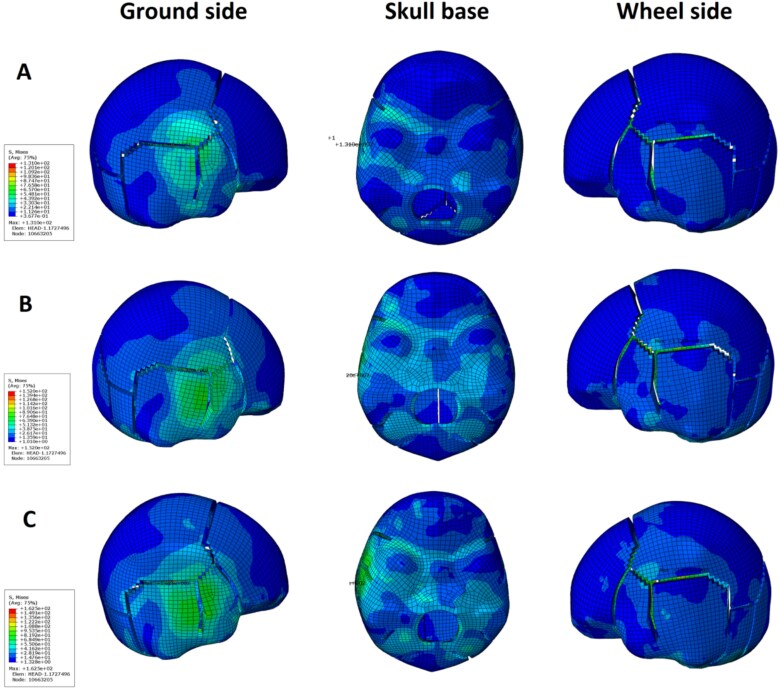
The loads of groups A, B, and C are 1 000 kg, 1 500 kg, and 2 000 kg, respectively. The maximum stress values, i.e. 131.0 MPa, 146.9 MPa, and 152.0 MPa, all occurred at the ground side of skull, and was spread roughly along the bone seam. The stress wave of the skull base propagated to the contralateral side generally along the corresponding position of the sphenoid wing and the arcuate eminence. With increasing load, the stress around the foramen magnum became more concentrated.

According to an actual case, medium vehicles caused multiple skull fractures, so the load applied to the head was set as 1 000 kg, 1 500 kg, and 2 000 kg. As a result, with increasing load, the stress in all parts of the skull showed an overall upward trend. However, the maximum stress values, i.e. 131.0 MPa, 146.9 MPa, and 152.0 MPa, all occurred on the ground side of skull. As shown in Figure 3, when the left and right temporal parts of the skull were crushed, the maximum stress was concentrated on the ground side of skull and was spread roughly along the bone seam. The stress wave of the skull base propagated to the contralateral side generally along the corresponding position of the sphenoid wing and the arcuate eminence. With increasing load, the stress around the foramen magnum became more concentrated.

## Discussion

With the development of virtual anatomy, CT plays an increasingly important role in forensic medicine, particularly in relation to violent injuries. The importance of CT in the diagnosis of head injury is evident [16–18]. However, due to its high cost, CT is rarely utilized during autopsies. Therefore, the cases we collected were not examined using CT but rather through anatomical methods.

In China, in addition to falling on the ground and being run over by a vehicle due to drunkenness or illness, there are more secondary accidents, including hit and runs. The secondary accident in this paper is defined as an accident in which the injured or dead person is hit or run over again by a vehicle after a road traffic accident according to the “Examination of Corpse in Road Traffic Accident GA/T 268-2019” standard [19] issued by the Ministry of Public Security of China in 2019. This study shows that riders were run over because the motor vehicle grazed a motorcycle or bicycle moving along a motor road, which is the most common cause of being run over, especially for electric bicycles [20, 21]. The second common cause is that a heavy vehicle or a medium vehicle, when turning right, knocked over pedestrians, bicycles or motorcycles, and then run over them. The reason is that because there is a blind spot on the right side of the vehicle, because the driving position of the vehicle in China is on the left side and along the right side of the road, and the driving position of heavy vehicles or medium vehicles is higher off the road. The third common cause is drunk people falling on the road and being run over. The fourth common cause is heavy and medium vehicles running over pedestrians, bicycles and motorcycles at intersections. The fifth common cause is disease, with just two cases. One is a mentally ill person sleeping on the road, and other is a person collapsed on the road after suffering a sudden brain haemorrhage. Amongst all causes, hit-and-run accounted for the largest proportion in cases where individuals were drunk and then run over. Consideration may be related to the driver trying to escape responsibility.

For secondary accidents of running over, forensic pathologists usually need to carefully identify the major injuries in the first accident and the second accident by systematic dissection combined with the traces found on the scene and the vehicle involved in the accident. In China, due to the frequent occurrence of secondary accidents and escape incidents, the Chinese government revised the “Examination of Corpse in Road Traffic Accident GA/T 268-2019” standard in 2019 [19], which further supplemented the contents of the systematic anatomy, understanding of the case, and understanding of the scene and vehicle traces. This study shows that each tyre of a heavy vehicle exerts ˃3 000 kg of pressure on the ground, often causing severe injuries to multiple organs after running over, as well as skull fractures with overflow of brain tissue. Amongst the nine cases of medium vehicles, there were two cases of skull fractures with brain tissue overflow. The tyres that ran over the heads exerted weights of 2 444 kg and 2 333 kg, respectively. Multiple skull fractures were found in seven cases of them, and the weight of the tyre ranged from 1 013 kg to 1 669 kg when the vehicle ran over the head. The cause of death from being run over by medium vehicles often results in severe damage to multiple organs. There were only two cases of skull fracture in a light vehicle and a light minicar, both of which were cases of falling down whilst riding a bicycle after drinking. Combined with on-site inspection, accident vehicle inspection and autopsy, it was confirmed that during falling, when the head collided with the ground, and the skull was fractured, after which being run over aggravated the degree of skull fracture. According to the measured weight of the tyre on the ground, no skull fracture occurred up to 941 kg, which differs from the findings of Yoganandan et al. [8]. Skull fractures are not only related to the magnitude of force but also to the location of impact, duration of the force, area of contact between the object causing injury and other factors. In this study, there were seven cases of multiple skull fractures caused by running over, all of which were caused by medium vehicles. Fractures occurred in both the tyre side and the ground side, and the ground side fracture was more serious than the tyre side fracture in all cases. The FE simulation showed that the stress on the skull was higher on the ground side than on the tyre side, and it decreased from the ground side to the tyre side along corresponding parts of the sphenoid wing and arcuate eminence. The FE simulation results confirmed that the ground side of head is more prone to fracture than the tyre side of head when the left and right temporal parts of the head are run over. Analysts believe that the main component of a tyre is rubber, which is more elastic than the ground. As a result, there is greater pressure on the ground and an increased likelihood of skull fracture due to the larger contact area between the tyre and head compared to that between the ground and head. In addition, tyre width, tyre pattern and tyre pressure may affect the occurrence of skull fracture. This study suggests that when a motor vehicle runs over the trunk of an individual first and then the left and right temporal parts of the head, light minicars and light vehicles usually do not cause skull fractures. If a skull fracture occurs, attention should be given to the possibility of it happening before being run over. Head injuries caused by a medium-sized vehicle running over someone are mainly characterized by multiple skull fractures, with more severe fractures occurring on the ground side than on the tyre side.
